# Ant Colony Optimization-Driven Ensemble Learning for Carbon Emission Modelling in Fly Ash–Slag Geopolymer Concrete

**DOI:** 10.3390/ma19102168

**Published:** 2026-05-21

**Authors:** Indra Kumar Pandey, Sanjay Kumar, Brajkishor Prasad, Pramod Kumar, Mizan Ahmed, Ardalan B. Hussein

**Affiliations:** 1 Department of Civil Engineering, National Institute of Technology, Jamshedpur 831013, India; 2 Department of Civil Engineering, Graphic Era Deemed to be University, Dehradun 248002, India; 3 Centre for Infrastructure Monitoring and Protection, School of Civil and Mechanical Engineering, Curtin University, Kent Street, Bentley, WA 6102, Australia; 4 Department of Structural Engineering and Geotechnics, Széchenyi István University, Egyetem Tér 1, 9026 Győr, Hungary

**Keywords:** geopolymer concrete, machine learning, carbon emissions, fly ash, ground granulated blast furnace slag, ensemble learning

## Abstract

This study investigates the prediction of carbon emissions from fly ash and ground granulated blast furnace slag-based geopolymer concrete (GPC) using advanced ensemble machine learning (ML) techniques. Although ML has been extensively utilized to model GPC’s mechanical performance, its application in estimating environmental impacts, specifically carbon emissions, is limited. The research employs six ensemble ML models, such as random forest, gradient boosting, extreme gradient boosting (XGB), CatBoost, and light gradient boosting machine (LGBM), including versions optimized using ant colony optimization (ACO). Among them, the ACO-enhanced XGB model demonstrated the highest predictive accuracy with a coefficient of determination (R^2^) of 0.97, with low prediction errors (MAE = 3.92, RMSE = 6.17). However, cross-validation and uncertainty analyses indicate that the performance differences among top models are relatively small. Conversely, LGBM exhibited the least predictive reliability. Feature importance analysis revealed that curing parameters, specifically initial curing time, curing temperature, and the dosage of dry sodium hydroxide, had the most influence on carbon emissions. To evaluate model robustness and interpretability, Monte Carlo simulation and Gaussian white noise analyses were conducted. Results confirmed that CatBoost and ACO–gradient boosting (ACO-GB) demonstrated greater stability under varying and noisy conditions, whereas XGB-based models, although highly accurate, were comparatively more sensitive to input variability. Overall, the research establishes a data-driven, efficient framework for quantifying carbon emissions in GPC, highlighting the importance of evaluating both predictive accuracy and model robustness, advancing sustainable material design through intelligent modelling.

## 1. Introduction

Concrete is the most widely used construction material globally due to its numerous advantages [1]. Ordinary Portland cement (OPC), as a primary ingredient of concrete that acts as a binder, emits 5–8% of total global CO_2_ emissions [2]. In addition to global warming concerns, the use of raw materials in OPC production depletes precious natural resources, causing ecological imbalance. To produce one ton of OPC, approximately 1.7 tons of raw materials are utilized [3]. Therefore, there is an imperative need to either reduce OPC consumption or eliminate it as a binder in concrete. With a rise in advocacy regarding the environmental threats posed by OPC, multiple studies have been conducted in the past as an attempt to replace OPC partially or completely with supplementary cementitious materials (SCMs) such as fly ash (FA) [4], ground granulated blast furnace slag (GGBFS) [5], rice husk ash (RHA) [6], etc. SCMs not only improve the strength and workability of concrete but also reduce CO_2_ emissions and mitigate the adverse environmental impacts associated with concrete production [7].

Among the available sustainable alternatives, geopolymer concrete (GPC) has emerged as a promising substitute for OPC. Geopolymers can be described as cementitious material produced from the reaction of alkaline activators with inorganic materials rich in silica (Si) and alumina (Al). Alkaline activators used for synthesizing geopolymers are usually a well-defined combination of hydroxyls (NaOH or KOH) and a glassy silicate, generally sodium or potassium silicate [8]. The process of geopolymerization creates tetrahedral aluminosilicate frameworks that provide sufficient strength for GPC. Furthermore, the geopolymerization process is also influenced by other factors, such as the type and amount of alkali activators, plasticizers and curing conditions [9]. The aforementioned factors have complex interrelationships and are major contributors to carbon emissions in GPC production.

To date, there has been a significant contradiction among researchers’ findings regarding the CO_2_ emissions of GPC compared with OPC concrete with the same binder content. Some studies have suggested that GPCs have almost 80% lower carbon emissions than OPC-based concrete [10]. On the other hand, findings of some scholars suggested a reduction ranging from 26% to 45% [11,12,13]. Furthermore, in some cases, OPC-based systems have 9% more emissions in comparison to GPC [14]. These noticeable differences are due to significant energy expenditure in manufacturing alkali activators and superplasticizers, and to high energy consumption during elevated-temperature curing.

Currently, most researchers are adopting experimental procedures to optimize the properties of constituent materials in GPC to maximize its performance [15,16,17,18,19,20]. However, conclusions drawn from these experimental procedures often have little potential to yield a generalized, accurate solution for estimating CO_2_ emissions. Furthermore, the amount and type of constituent materials play a significant role in determining the CO_2_ emissions from GPC [21]. Therefore, instead of using time- and labor-intensive experimental procedures, empirical methodologies can be adopted to predict CO_2_ emissions. However, empirical equations cannot capture intrinsic data relations and are unable to produce a generalized and effective solution. Furthermore, recent technological advancements can leverage machine learning (ML) algorithms to address these complex issues. The development of sophisticated ML-based algorithms has great potential to address the complex relationships between constituent materials and GPC CO_2_ emissions.

In recent years, ML has gained widespread attention from researchers in different research domains, such as science [22,23,24], construction [25], engineering [26,27], medical [28,29], and business analytics, because of its simplicity and accuracy. Multiple ML approaches have been adopted by various researchers to estimate various properties of concrete. For instance, Gomaa et al. [30] applied the random forest algorithm with optimized hyperparameters for the prediction of slump flow and compressive strength of FA-GPC by including the chemical composition of constituent materials. Zhang et al. [31] enhanced compressive strength prediction of GGBFS and fly ash-based alkali-activated materials by incorporating five chemistry-informed features in a GBM model trained on 676 data points. For MK-based geopolymers, Lahoti et al. [32] classified strength into low, medium, and high categories using a dataset of 71 instances, analyzing key mix design parameters. However, their ML models struggled to achieve accurate predictions.

In addition to standalone ML algorithms, hybrid models have been developed by multiple researchers to improve model accuracy. For instance, Rachel et al. [33] proposed a hybrid ML model that integrated a random forest with the firefly algorithm to enhance the predictive performance for concrete compressive strength. Their findings demonstrated that the proposed hybrid model outperformed standalone ML algorithms such as support vector machines and random forests. Shariati et al. [34] also proposed a hybrid ML model using the grey wolf optimizer to forecast concrete compressive strength with partial cement replacement. The results were compared with ANFIS, ELM, ANN, and SVR models using RBF and polynomial kernels.

Furthermore, only two studies are available for predicting GPC carbon emissions using ML algorithms, to the authors’ best knowledge. For instance, Wudil et al. [35] proposed three ML models, viz. Gaussian process regression (GPR), support vector regression (SVR), and standalone decision tree regression (DTR) to predict carbon emissions of FA-GPC with seven input parameters in 832 data rows. The results of this study demonstrated the efficiency of ML models to accurately predict carbon emissions. However, this study did not account for the effects of curing duration, water content, and NH molarity on carbon emissions. Furthermore, a similar study was conducted by Al-Fakih et al. [36] to predict the carbon emissions of GGBFS-GPC using stacking ensemble techniques on a chemistry-informed dataset comprising 131 rows. It was concluded that model selection should be based on the characteristics of the dataset. Their study also concluded that stacked ensemble techniques are effective for predicting carbon emissions. Superplasticizer, curing temperature, and dry NaOH content were the most influential factors in the prediction of CO_2_ footprint.

Beyond traditional civil engineering applications, geopolymer materials have increasingly been explored in subsurface and resource engineering domains due to their superior physicochemical stability and durability under aggressive conditions. Their inherent resistance to high temperatures, low permeability, and chemical inertness make them suitable for geothermal energy systems, particularly in mine-based geothermal extraction, where materials must withstand coupled thermal, hydraulic, and chemical effects. Recent studies have indicated that geopolymer-based binders can enhance wellbore integrity and sealing efficiency in such environments [37]. In addition, geopolymers have shown significant potential in sustainable mining operations, especially in coal mine backfill applications. Their compatibility with industrial by-products and ability to develop adequate mechanical strength make them viable alternatives to conventional cementitious binders in cemented rockfill systems. The incorporation of advanced modifiers, such as cellulose-based dispersing agents, further improves the homogeneity and strength characteristics of these backfill materials [38]. These emerging applications highlight the broader interdisciplinary relevance of geopolymer technology and support its continued development for sustainable infrastructure and resource management.

Despite growing interest in sustainable construction materials, very little research exists on predicting GPC carbon emissions using ML techniques. While previous studies have attempted to estimate carbon emissions, a significant research gap persists. Despite huge potential as a sustainable aluminosilicate material, there is a scarcity of research focused on predicting carbon emissions of FA-GGBFS-GPC [39]. Furthermore, developed models in existing literature lack generalizability and robustness due to the utilization of relatively small datasets. The scarcity of data hinders the reliability of models in real-world applications.

While geopolymer systems are widely recognized as sustainable alternatives to OPC-based materials, the quantification of their carbon emissions remains highly inconsistent in the literature. Reported reductions vary significantly due to the complex, nonlinear interactions among multiple parameters, including precursor composition, alkaline activator dosage, curing regime, and mix design variability. Existing approaches, which are primarily based on simplified empirical or heuristic formulations, are limited in their ability to capture these multidimensional interactions and often lack generalizability across datasets and production conditions.

In this context, the present study does not merely re-examine the low-carbon nature of geopolymer materials but aims to develop a robust and generalizable predictive framework for estimating carbon emissions. The novelty of this work lies in several key aspects. First, ensemble machine learning models are systematically hybridized with ant colony optimization (ACO) to enhance predictive capability through optimal hyperparameter selection. Second, a comprehensive and relatively large dataset comprising 1341 data points is utilized, which improves model reliability compared to prior studies based on limited datasets. Third, the modelling framework explicitly incorporates process-sensitive variables, such as curing temperature, curing duration, and alkaline activator characteristics, enabling a more realistic representation of emission sources. Fourth, model robustness is rigorously evaluated through Monte Carlo simulations and Gaussian white noise analysis, providing insights into stability under uncertainty. Finally, feature importance analysis is employed to interpret the model’s behavior and identify the dominant parameters influencing carbon emissions, thereby supporting informed and sustainable mix design. Various statistical performance indicators, including R^2^, RMSE, MSE, RSE, and the a20-index, will be used to evaluate the models’ predictive performance.

Therefore, the contribution of this study lies in advancing carbon emission modelling of geopolymer concrete from conventional deterministic estimation towards a data-driven, interpretable, and uncertainty-aware predictive approach.

## 2. Database Creation and Data Preparation

### 2.1. Data Collection

The quality of data directly influences the performance and accuracy of machine learning models, as these models completely depend on the information supplied to them. Poor-quality data can result in unreliable and inconsistent model performance. To ensure good-quality data for the present investigation, the following benchmarking criteria were adopted:

- Data was extracted from reputed, peer-reviewed journal articles.
- Detailed information about the mix proportions for FA-GGBFS-based geopolymer concrete (GPC) was available.
- Only GGBFS produced from steel manufacturing industries and FA from thermal power plants were considered for the present investigation, excluding any other aluminosilicate materials.
- Studies with proper descriptions of the initial curing conditions, including time and temperature, were included.
- The amount of additional water added, if applicable, needed to be clearly mentioned in the data source.

Based on the above benchmarking criteria, a total of 1341 data points were collected [40,41,42,43,44,45,46,47,48,49,50,51,52,53,54,55,56,57,58,59,60,61]. To avoid the risk of errors during the prediction phase, the collected data was then cleaned by handling missing and duplicate values. Furthermore, to improve data reliability, outlier treatment was performed.

Since the collected database was compiled from multiple published studies, a systematic data harmonization procedure was performed prior to model development. All variables were converted into a consistent unit system to ensure inter-study compatibility. Material quantities were standardized to kg/m^3^, curing temperature to °C, curing duration to days, and calculated carbon emissions to kg CO_2_/m^3^. Studies containing incomplete mix design information, unclear curing conditions, inconsistent reporting formats, or insufficient emission-related parameters were excluded from the database. Missing values for critical input variables were not imputed; instead, such records were removed to preserve the dataset’s reliability and avoid introducing artificial bias into the machine learning models. Duplicate entries resulting from overlapping datasets or repeated reporting were identified and eliminated. In addition, data consistency verification and outlier screening were conducted to minimize anomalous observations and improve the robustness and generalizability of the predictive models developed.

### 2.2. Calculation of CO_2_ Emissions

The carbon emission assessment boundary was limited to a cradle-to-gate approach, which includes emissions generated from raw material extraction, processing, constituent material production, and thermal curing processes associated with FA-GGBFS-based geopolymer concrete. Emissions related to transportation, onsite construction activities, operational service life, demolition, recycling, and end-of-life disposal were excluded due to the lack of consistent and reliable information in the collected database. Therefore, the calculated CO_2_ emissions represent only the material production- and curing-related environmental impacts.

**Figure 1:**
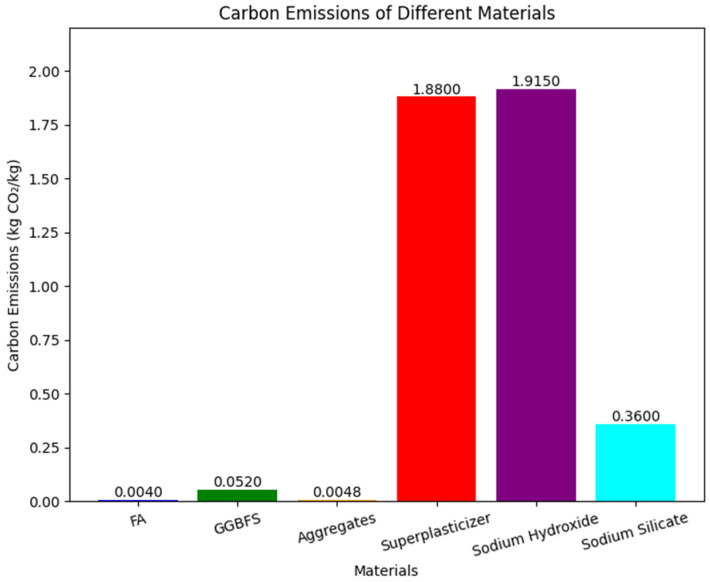
CO_2_ emissions per unit of constituent materials in FA-GGBFS-based GPC in kg of CO_2_ per kg of constituent materials [36].

The CO_2_ emissions for FA-GGBFS-GPC include emissions (Equation (1)) generated from the production and extraction of each constituent raw material [43], as well as from the curing temperature. These factors are integrated together by using a heuristic equation proposed by Yang et al. [61].

(1) $$Carbon\;Emissions\;\left(kg\;{CO}_{2}/m^{3}\right)=\sum_{i}{(w}_{i}\times m_{i})+\left(0.642T-16.042\right)\times t$$

where $w_{i\;}$= carbon produced from the production of one ton of constituent material *i*, obtained from Figure 1; $m_{i}\;$= mass (t/m^3^) of constituent material *i* in fresh mix; *T* = curing temperature (°C); and *t* = days of curing.

There are two components in the equation above used to calculate carbon emissions for FA-GGBFS-GPC. The first component determines the total emissions by summing the CO_2_ footprints of each material in the GPC, weighted by their respective masses (*m_i_*). The second component of the equation integrates the effect of heat curing. In this part, the curing duration (t) was considered only when the curing temperature (T) exceeded 25 °C; otherwise, it was set to zero.

However, it should be acknowledged that the adopted CO_2_ estimation methodology is based on a simplified heuristic formulation and therefore has limitations that may affect the reliability and generalizability of the predicted emissions. Although the adopted cradle-to-gate system boundary captures emissions associated with raw material extraction, constituent production, and thermal curing, it excludes transportation-related emissions and variations arising from differences between laboratory-scale specimen preparation and industrial-scale production systems. In practical industrial applications, factors such as transportation distance, energy source, plant efficiency, material handling procedures, and large-scale processing conditions may substantially alter the overall carbon footprint of geopolymer concrete. Consequently, the calculated CO_2_ values should be interpreted as approximate estimates within the defined assessment boundary rather than absolute lifecycle emission values. These simplifications may introduce uncertainty into the dataset used for machine learning model development and can affect the direct applicability of the developed models to full-scale industrial scenarios. Nevertheless, because a consistent estimation framework was applied uniformly across all data samples, the developed ML models remain effective at identifying relative emission trends and capturing the nonlinear relationships among mixture parameters, curing conditions, and associated carbon emissions. Future studies should incorporate transportation effects, industrial production datasets, and comprehensive lifecycle inventory data to improve the robustness and real-world applicability of the proposed predictive framework.

It is worth noting that the heuristic equation used to compute CO_2_ for the GPC mixture is based on the methodologies of Breiman [62]. To improve the accuracy of the proposed equation, a linear regression analysis was applied to estimate CO_2_ emissions from heat curing, following the guidelines proposed by Wu et al. [63]. However, this model does not include emissions from material transportation, and, due to the absence of relevant data, it also does not account for variations between large-scale industrial production and controlled laboratory environments. Although previous studies [62,63] suggested that the equation achieves reasonable accuracy in estimating CO_2_ footprints, further research about the lifecycle analysis of FA-GGBFS-GPC is necessary to provide more validation for this model. Such an approach would offer a more comprehensive understanding of the equation’s reliability in different conditions and production systems.

### 2.3. Description of the Dataset

The selected input parameters for the machine learning model include FA, GGBFS, fine aggregate, coarse aggregate (CA), total aggregate (TA), sodium silicate (NS), sodium hydroxide (NH) liquid, molarity (M), NH dry, added water, superplasticizer (SP), total water (TW), initial curing time (ICT), temperature (temp), and initial curing rest time (ICTRT). These parameters were chosen based on their contribution to carbon emissions during the production of FA-GGBFS-GPC. The CO_2_ emissions, calculated using the equation in Section 2.2, serve as the output variable.

**Figure 2:**
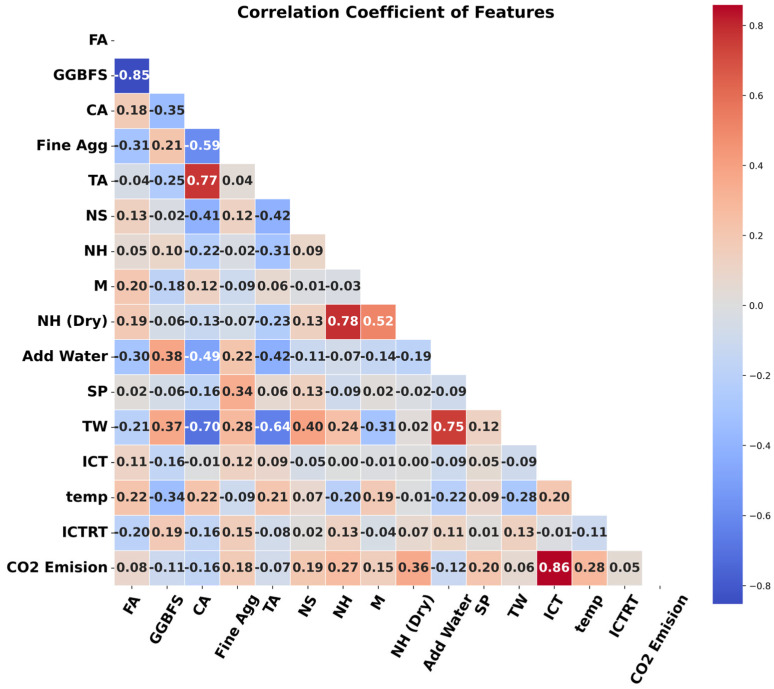
Correlation matrix for data distribution.

**Figure 3:**
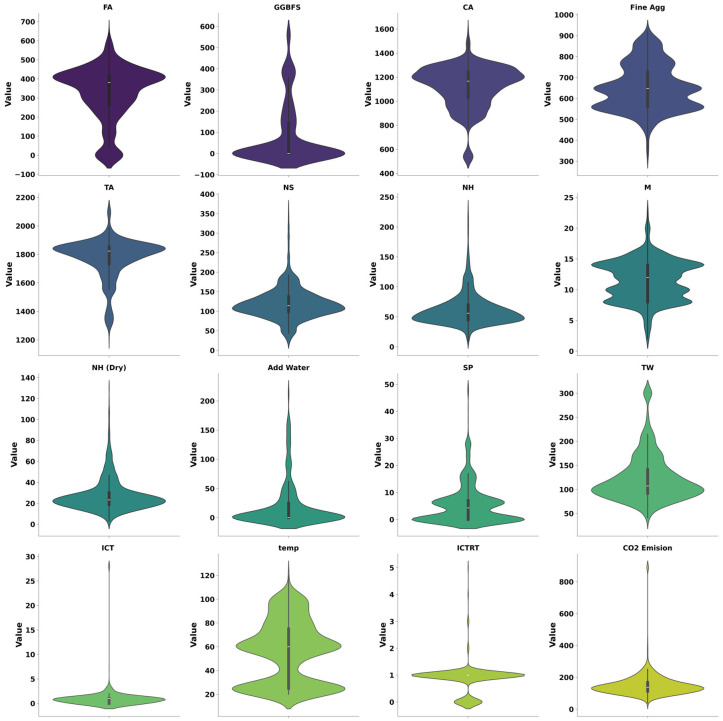
Violine plot for data distribution.

Table 1 presents a detailed statistical analysis of the distributions of all input and output variables. This analysis includes key statistical measures such as data count, mean, standard deviation, minimum and maximum values, first quartile, median (middle quartile), and upper quartile, ensuring the dataset’s consistency and stability for use in this study. In addition to statistical analysis, a correlation analysis between input and output variables, as well as among the input variables themselves, is illustrated in Figure 2. This analysis provides insights into how each input variable influences CO_2_ emissions. The correlation coefficient was used to assess the sensitivity of each FA-GGBFS-based GPC parameter to CO_2_ emissions. The heat map reveals that GGBFS, coarse aggregate, total aggregate, and added water are negatively correlated with CO_2_ emissions, whereas the remaining input variables are positively correlated. A positive correlation indicates that increases or decreases in these input parameters result in corresponding changes in CO_2_ emissions. Conversely, negatively correlated variables are inversely proportional to the output, meaning an increase in these parameters leads to a decrease in CO_2_ emissions. Furthermore, Figure 3 provides a comprehensive understanding of data distribution and variability.

From Figure 3, it is evident that FA, GGBFS, fine aggregate, CA and TA possess distinct density patterns. FA and TA have relatively stable distributions, indicating their consistent presence in the data. The multimodal distribution of GGBFS indicates its use in specific GPC mixes. NS and NH also demonstrated multimodal distribution. The density spread suggests a preference for specific combinations in GPC. However, the NH (Dry) plot is slightly skewed, revealing its concentration on lower values, with occasional higher values. The irregular distribution of M, with multiple peaks, reflects variations in alkaline concentration. High-density clusters in SP distribution suggest the frequent use of standard doses. Furthermore, the distribution of ICT is skewed, with lower values being more frequent, and ICT exhibits a dispersed pattern. In addition, the CO_2_ emission plot shows a right-skewed distribution, indicating that high CO_2_ emissions occur less frequently. A total of 70% of the collected data was used for training the models, and the remaining 30% was utilized for testing the model performance. Furthermore, to assess the reliability of each model’s output, 10-fold cross-validation was performed. While feature importance analysis provides a comparative ranking of influential variables across different models, it does not capture the direction and magnitude of their contributions to model predictions. Therefore, to gain a more comprehensive understanding of model behavior, SHAP (SHapley Additive exPlanations) analysis was employed.

## 3. Machine Learning Algorithms

### 3.1. Random Forest

Random forest (RF) is an ensemble ML technique that combines the outputs of multiple decision trees (DTs) to improve accuracy and robustness [63]. Each tree in RF is trained on a randomized data subset to mitigate overfitting and enhance generalization performance. It can handle many input parameters with minimal performance loss, making it suitable for high-dimensional data. Feature randomization during the training process acts as a built-in instrument for feature selection, as less prominent variables have less probability to be selected consistently across the trees. Moreover, RF uses the Gini impurity criterion to compute the participation of each feature to bring down the impurity of predictions [64]. When handling complex, noisy data, RF can be a reliable method for regression and classification. It offers greater stability than single DTs by averaging predictions and reducing prediction variance [65]. Furthermore, this approach is highly resilient to outliers, as the impact of noisy data is diluted across multiple DTs [66]. RF has a self-assessment nature for further performance improvement through out-of-bag error estimation, which facilitates an intrinsic measurement of accuracy during the training phase. This abolishes the need for separate validation datasets to verify model accuracy. As a result, RF can be considered a powerful and efficient algorithm for handling high-dimensional, complex data with high precision and interpretability [67].

### 3.2. Gradient Boosting

Gradient boosting regressor (GBR) is a versatile ensemble learning algorithm, known for its ability to capture complex relationships in the data [68]. A stagewise additive perspective is used by GBR to optimize a differential loss function. For each iteration, GBR fits a classification and regression tree (CART) with the negative gradient of the loss function, which is a representation of residual error between forecasted and real values [69]. GBR minimizes the risk of overfitting by effectively handling noisy data, thus producing robust predictions. Moreover, GBR models are interpretable because they can estimate the relative importance of input features used during training. The GBR model has two intrinsic characteristics that influence its performance: complexity and generalization ability. One is the number of boosting iterations, and the other is the shrinkage parameter (learning rate) [70]. Generally, GBR models start with a tree having only one leaf node, i.e., γ, that minimizes the loss function over all the samples with the help of Equation (2).

(2) $$F^{0}\left(x\right)={argmin}_{\gamma }\sum_{i=1}^{n}L(y_{i},\gamma )$$

This is followed by multiple iterations to compute the negative gradient of the loss function L, which will subsequently be used to train a DT, and a new model will be added to the ensemble. This process can be expressed by using Equation (3).

(3) $${F\left(x\right)=F^{m-1}\left(x\right)+µf}^{m}(x)$$

where µ is used to control overfitting and is known as a shrinkage parameter. Although GBR is used for regression analysis in the present investigation, it can also be used for classification tasks. More fundamental theoretical details for GBR can be found in [68].

### 3.3. Extreme Gradient Boosting Machine Regressor

The extreme gradient boosting machine (XGB) is a powerful ensemble learning technique that is well suited to non-linear and multivariate function approximation tasks [71]. It continuously refines a weak regression tree by incorporating additional models that focus on reducing the prediction errors of previous iterations. This process yields a powerful ensemble that reduces both bias and variance. A gradient descent optimization is formulated in the learning phase over an explicit objective function to ensure improved predictive performance with each added model. The following mathematical expression (Equation (4)) can be used to define the objective function in the XGB model [72].

(4) $$f_{obj}=\sum_{i}L(y_{i},\;F(x_{i}))+\sum_{k}\Omega \left(f_{k}\right)\;$$

where

$\Omega \left(f\right)$ *=* $\gamma T+\;\frac{1}{2}\lambda \sum_{j}^{T}\omega_{j}^{2}$ is known as a regularization term.

T is the number of leaves in a regression tree $f_{i}$.

It is mandatory to specify a loss function (*L*) to calculate $f_{obj}$. Squared error loss is a frequently used loss function for regression problems, which can be computed using Equation (5) [73].

*L* (*t*, *y*) = (*t* − *y*)^2^ (5)

where

*t* and *y* are the actual and forecasted values of the response, which is CO_2_ emission in the present study.

Taylor’s second-order approximation is used to arrive at the solution to the above-mentioned optimization problem [74]. Furthermore, the loss function L (t, y) must be computed for the first- and second-order gradients. According to this, the optimal score for each DT can be calculated using Equation (6).

(6) $$\omega_{j}^{*}=-\frac{\sum_{i\in I_{j}}g_{i}}{\sum_{i\in I_{j}}{(h}_{i}+\lambda )}$$

where $g_{i}$ and $h_{i}$ are the first-order and second-order gradient of *L*(*t*, *y*), and the data instances that reach a leaf are denoted by *I_j_*. After successful training, the final model for output estimation is given by Equation (7).

(7) $$F_{XGB}=\sum_{k=1}^{M}f_{k}(x)$$

where x is the feature vector of the model, which describes the characteristics of HPC mixes; M denotes the number of individual models in the ensemble; and F_XGB_ is the CS value of the mix estimated by XGB.

To ensure improved predictive performance, this algorithm takes advantage of an advanced tree-splitting mechanism, which is complemented by sample randomization to introduce heterogeneity among trees for the creation of a barrier against model overfitting and to ameliorate generalization ability. The level-wise strategy for feature-split evaluation also improves XGB’s power, allowing it to systematically learn from complex data relationships. One of the critical advantages of XGB is its parallel processing capability, leveraging the computational power of graphics processing units to handle large, high-dimensional datasets.

### 3.4. LightGBM Regressor

Traditional gradient-boosted decision tree (GBDT) algorithms require an exhaustive, computationally intensive process of data scanning to compute information gain at prospective split points for each node. This limits the efficiency and scalability of traditional GBDTs, and to resolve this issue, LGBM was introduced by Microsoft with two novelties: gradient-based one-side sampling (GOSS) and exclusive feature bundling (EFB). The GOSS technique focuses only on samples with large gradient values, treating them as the most significant parameter for calculating information gain during split-point selection [75]. By eliminating a substantial portion of data samples with low gradients, GOSS significantly reduces computational time while accurately calculating information gain. If *k_i_* denotes the gradient of sample i, the objective loss function for boosting trees in GBDT can be expressed as in Equation (8).

(8) $$latinL=\sum_{i=1}^{n}l(y_{i},\;\hat{y_{i}})$$

where l is a loss function, and $y_{i}$ and $\hat{y_{i}}$ are the actual and predicted values, respectively. In each iteration, GOSS preserves a subgroup of samples with the largest gradients and a random subset of the remaining samples with smaller gradients [76]. The preserved subgroup is utilized in the computation of information gain (IG) for the selection of splitting points, which can be expressed as Equation (9).

(9) $$IG=\frac{1}{2}(\frac{{\left(\sum_{i∊L}k_{i}\right)}^{2}}{\left|L\right|}+\frac{{\left(\sum_{i∊R}k_{i}\right)}^{2}}{\left|R\right|}-\frac{{\left(\sum_{i∊P}k_{i}\right)}^{2}}{\left|P\right|})$$

where the parent node is denoted by P, and the left and right child nodes are denoted by L and R, respectively. On the other hand, the EFB technique addresses the NP-hard problem of bundling mutually exclusive features (which rarely have non-zero values simultaneously) by reducing them to a smaller set of composite features [77]. This dimensionality reduction enhances computational efficiency while preserving the integrity of the split-point identification process. Though LGBM is utilized for regression tasks in the present study, it is also suitable for all kinds of supervised learning problems.

### 3.5. CatBoost Regressor

Traditional gradient boosting algorithms struggle to effectively handle categorical features [78]. The CatBoost regressor (CBR) algorithm is designed to address the limitations of traditional GBDT models with its robust capability for handling categorical features. This advanced ML algorithm includes two core innovations: ordered boosting and an advanced categorical feature handling method. Ordered boosting is a traditional gradient-boosting variant that is permutation-driven and specifically tailored to handle prediction bias caused by target leakage [79]. Ordered boosting ensures that the gradient of each data point is computed without adopting its own target value by utilizing multiple permutations for data splitting.

CBR leverages sophisticated encoding methods to handle categorical features, replacing traditional one-hot encoding. It uses statistical approaches to replace the value of a categorical feature with the average target value conditioned on that feature’s value [80]. To avoid the risk of overfitting, a Bayesian smoothing technique is adopted to regularize this process. CBR also employs a novel schema to compute leaf values when selecting the tree structure. It optimizes the leaf value and penalizes the overfitting with the help of a target function. This process improves the model’s predictive accuracy while providing robust control over overfitting.

### 3.6. Ant Colony Optimization

Ant colony optimization (ACO) was first proposed by Dorigo in 1990 [81]. ACO is a swarm intelligence algorithm that mimics the foraging behavior of ants to solve optimization problems. It replicates the behavior of ants discovering the shortest path from a food source to the ant nest by laying pheromone markings. ACO operates on three major principles: pheromones are deposited along the path to the food source, motivating other ants in the colony to follow; paths with higher pheromone intensity are more attractive and more likely to be followed by other ants; and evaporation of pheromones prevents over-reliance on suboptimal solutions [82]. Ants collectively converge towards the most efficient path over successive iterations. This leads to optimal or near-optimal solutions.

ACO has been extensively used in the field of logistics, network routing, and task scheduling. It has also demonstrated strong potential in solving regression problems by optimizing influential parameters and minimizing error functions [83]. Its probabilistic and iterative refinement approach offers competitive results compared to other optimization techniques. In the present investigation, ACO was used as an optimization technique to find the best combination of hyperparameters in all ensemble learning models.

### 3.7. Model Performance Metrics

The performance of the ML algorithms applied in the present study was evaluated using various statistical metrics, including the coefficient of determination (R^2^), mean absolute error (MAE), residual standard error (RSE), root mean squared error (RMSE), relative root mean squared error (RRMSE), and a20. R^2^ provides insight into how well the model fits the data. If the value of R^2^ is close to 1, it indicates that the model explains most of the variation in the input data. In contrast, MAE computes the average error across a set of predictions without considering whether the predictions are overfitting or underfitting. A smaller MAE indicates that the model’s predictions are closer to the actual values.

Similarly, a lower RSE value indicates more consistent model performance that is tightly aligned with the actual values. Furthermore, RMSE indicates the magnitude of errors and is particularly sensitive to larger errors. Lower RMSE values signify that the model’s predictions are closer to the actual values. To increase the reliability of the developed model, another statistical parameter, i.e., a20, is used in the current study. It measures the fraction of predictions that fall within a 20% margin of the actual values. Unlike RMSE and MAE, a20 places greater emphasis on relative accuracy than on an absolute measure of error. The mathematical expressions of all statistical metrics used in this study are represented in Equations (10)–(14) [75,76,80].

(10) $$R^{2}=1-\frac{\sum {\left(Y_{i}-{\hat{Y}}_{i}\right)}^{2}}{\sum {\left(Y_{i}-\bar{Y_{i}}\right)}^{2}}$$

(11) $$MAE=\frac{1}{n}\sum \left|Y_{i}\right.-{\hat{Y}}_{i}|$$

(12) $$RSE=\sqrt{\frac{\sum {\left(Y_{i}-{\hat{Y}}_{i}\right)}^{2}}{n-p}}$$

(13) $$RMSE=\sqrt{\frac{1}{n}\sum {\left(Y_{i}-{\hat{Y}}_{i}\right)}^{2}}$$

(14) $$a20=\frac{\sum Ɉ(|Y_{i}-{\hat{Y}}_{i}|\leq 0.2Y_{i})}{n}\times 100$$

where $Y_{i}\;$= actual observed value, ${\hat{Y}}_{i\;}$= predicted value, $\bar{Y_{i}}$ = mean of actual values, *n* = total number of observations, p = number of features present in model, and Ɉ = indicator function that counts the occurrence of a condition being met.

## 4. Results and Discussions

This section deals with outcomes of various machine learning algorithms for predicting the carbon emissions of FA-GGBFS-GPC. According to Ballabio et al. (2018) [73], the ratio of the number of rows in a dataset to the number of input parameters should be greater than 3, and preferably greater than 5 for a more robust model. In the present study, the ratio of the dataset size to the number of input parameters was 1340/15 > 5, indicating a favorable condition for robust fitness.

### 4.1. Hyperparameter Tuning

Hyperparameter tuning is an important procedure in ML models to optimize the performance of developed models. Appropriate selection of hyperparameter combinations improves the model’s accuracy and generalization ability. In this study, an optimal combination of all five ensemble learning models was identified using ACO. The selected hyperparameters and their values are presented in Table 2. The key hyperparameter n_estimators specifies the number of trees in an ensemble model. A higher number of estimators generally increases the model accuracy but also increases the computational time. Similarly, learning rate plays an important role in boosting models. It controls the step size while correcting model weights. Lower learning rates provide stable learning but require more computational time due to increased iterations [5,11]. However, a higher learning rate can increase triage speed but may lead to suboptimal solutions. Therefore, careful selection of the learning rate to balance model speed and accuracy is extremely important. Furthermore, maximum depth is another important hyperparameter that demonstrates the depth of trees in ML models. Deeper trees capture more complex relationships in the data but may produce overfitting in the model in the absence of proper regularization. Alongside this, the subsample demonstrates the fraction of the training data allocated to each tree, helping prevent overfitting by introducing randomness into the data. To further reduce the complexity of developed models, the minimum sample leaf and minimum sample split were adjusted for the RF and GBR algorithms. These hyperparameters determine the minimum number of samples required to split a node from a leaf.

To further improve model performance, regularization factors were optimized for CBR and LGBM models. Minimum child weight was optimized in CBR and GBR to ensure that each child node had enough data for better generalization. On the other hand, alpha and lambda as L1 and L2 regularization terms were tuned in LGBM to prevent excessive complexity while maintaining model flexibility. Additionally, the number of child samples and the number of leaves were optimized for the LGBM model to control the structure of the trees. Overall, each model was optimized for its strengths to ensure optimal model performance while preventing excessive complexity and inefficient computational costs.

### 4.2. Model Performance Comparison

**Figure 4:**
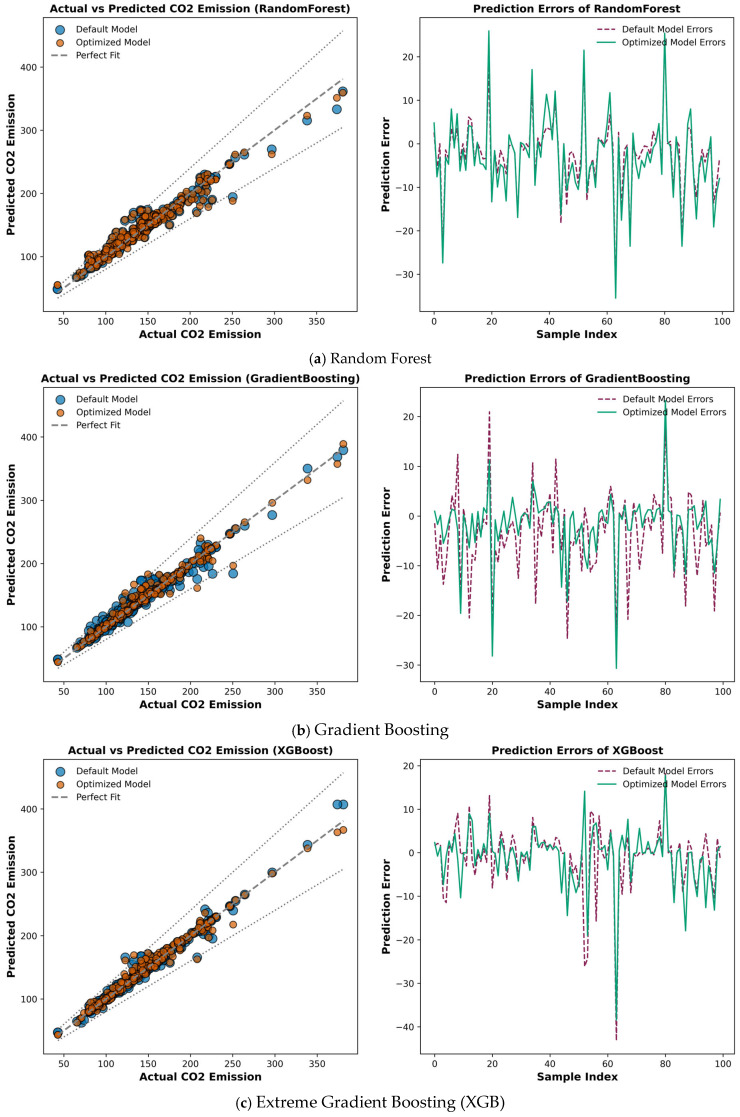

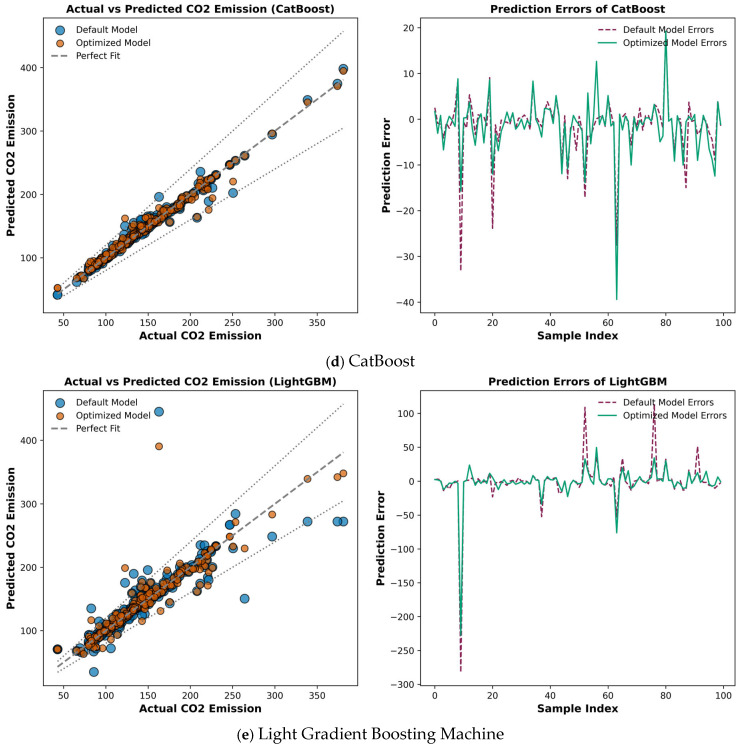
Model performance of all models on training and testing data.

**Figure 5:**
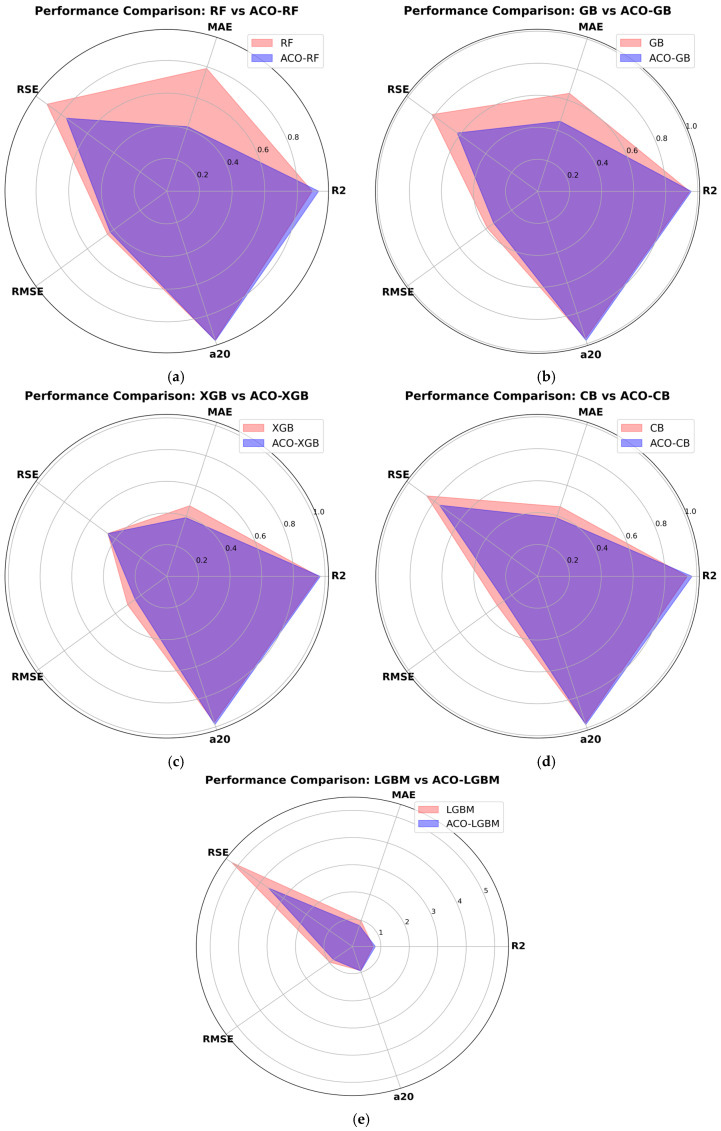
Performance comparison of ensemble and hybrid ensemble models.

Table 3 summarizes the results of statistical performance metrics of all the models and can be visualized in Figure 4. To further enhance the understanding of the results, Figure 5 presents a visual representation of the performance metrics for all models. XGB performed exceptionally well on the training data, achieving an R^2^ value of 0.98, indicating a good model fit and generalization ability on the training data. The test R^2^ (0.96) was slightly lower than the training R^2^, manifesting in a minor loss in the model’s generalization ability, which is common in ML algorithms. XGB demonstrated strong predictive performance on unseen test data for carbon emissions. The MAE and RMSE on the test dataset were 4.37 and 7.64, respectively, which are relatively low, indicating low overall prediction error and enabling XGB to make accurate carbon emissions predictions [60]. A lower RSE value (0.025) for the test data indicates that the squared error is low relative to the data’s variance.

ACO-XGB outperformed the XGB model across all metrics. The R^2^ values for the training and testing data were 0.99 and 0.97, respectively, slightly higher than, but nearly identical to, the XGB model. Furthermore, the higher R^2^ values in the training and testing data of ACO-XGB suggested that there was no detrimental impact of optimization on the model’s capabilities of capturing complex data trends. The reduction in MAE (3.9) and RMSE (6.17) of test data stated a clear accuracy improvement with reduced error after optimization. On top of that, the RSE value of the hybrid XGB model was 0.023 on the test set, slightly lower than XGB’s, indicating better performance for ACO-XGB.

The RF model performed slightly well, demonstrating effective model fitting on the training data, with an R^2^ of 0.96. However, the R^2^ on the test set increased to 0.89, indicating some degree of model overfitting and poor generalizability [55]. Moreover, the higher MAE (7.9) and RMSE (11.23) values for the test data indicated deviations between predicted and actual carbon emissions and revealed notable prediction errors. On top of that, the training and testing RSE values for the RF model were observed as (0.008) and (0.04), respectively. A higher RSE value for test data indicated the model’s inefficiency in terms of squared errors relative to data variance.

After hyperparameter optimization of the RF model, the ACO-RF hybrid model demonstrated improved performance over the default RF model. The value of test R^2^ (0.93) was improved by 4%, ameliorating the generalization ability of the ACO-RF model. Moreover, this significant improvement in R^2^ value indicates that the hybrid ACO-RF model efficiently captured intrinsic data patterns. Also, the MAE, RSE, and RMSE values of the test data were reduced to 4.15, 0.038, and 10.68, respectively, in comparison to the default RF model values, signaling in the direction of improved accuracy and reduced errors through the optimization process.

The results show that the R^2^ values for the training and testing data for GB were 0.97 and 0.95, indicating a reasonable ability to predict carbon emissions from FA-GGBFS-GPC. Though the lower test MAE (6.44) indicated good accuracy, GB’s RMSE (9.71) was slightly higher than XGB’s, and ACO-XGB demonstrated less precise carbon emission predictions. These statistical metrics are competitive but cannot outperform ACO-XGB.

The ACO-GB model achieved better performance metrics than GB. The R^2^, MAE, RSE, and RMSE values for the training data set were observed as 0.99, 1.59, 0.0009, and 2.58, respectively, and for the test data set, 0.96, 4.60, 0.031, and 8.46, respectively. ACO reduced MAE, RSE and RMSE in comparison to GB, demonstrating a more accurate model obtained by hyperparameter optimization. Despite notable performance improvements, ACO-GB still could not match the performance of the ACO-XGB and XGB models.

The training and testing R^2^ values for the CB model suggested a good fit to the training data and better generalization. The MAE and RMSE values for the CB training data were 1.46 and 2.46, respectively. Moreover, the corresponding test dataset values were 4.60 and 7.81, indicating accurate carbon emissions predictions, though not as accurate as those from XGB. The training and testing R^2^ values were 0.97 and 0.94, which were high but not as high as those of XGB and ACO-XGB, indicating that the model was slightly struggling to capture complex relationships in the data. Also, the RSE value in the test data was 0.0436, indicating moderate model performance, with slightly higher prediction errors than ACO-GB and ACO-XGB.

On the other hand, ACO-CB demonstrated performance improvement over the CB model. The training and testing R^2^ values of ACO-CB were observed as (0.99) and (0.97), respectively. The higher R^2^ values for the test data reflected an even more powerful capability of explaining variance in the dataset. The MAE, RSE, and RMSE values for the training data were 1.17, 0.0007, and 6.45, respectively. The MAE and RMSE values of the test data were (3.86) and (6.45), respectively. This demonstrated the strong predictive power and generalization ability of the ACO-CB model in predicting carbon emissions of FA-GGBFS-GPC [40]. Also, model optimization improved the model’s efficiency, yielding lower RSE values than the default CBR model.

LGBM illustrated the most muted performance among all the developed predictive models. The training and testing R^2^ values for the LGBM model were 0.97 and 0.73, indicating poor model generalization. The training MAE, RSE, and RMSE values were 5.37, 0.023, and 13.03, respectively. The MAE and RMSE values for the test data were 9.87 and 24.69, respectively, both much higher than their counterparts, resulting in substantial prediction errors. These values were much lower than those of all other models, reflecting the model’s poor ability to explain variance in the training and testing datasets. The RSE of LGBM was very high, with values of 0.023 and 0.26 for the training and testing data, respectively, indicating inefficiency in terms of squared error relative to the data variance.

ACO-LGBM demonstrated some improvement over the default LGBM model. The R^2^ values for the training and testing data were 0.98 and 0.81, respectively, both higher than the default LGBM model. This improvement signaled a significant upward shift in the model’s generalization ability. The MAE and RMSE were reduced to 8.01 and 20.55, respectively, although these values still indicated substantial error relative to the other models. The NSE value for the test data was 0.81, higher than the default LGBM model, indicating some improvement, but it was still much lower than all the other models [33]. The RSE value for the test data in the ACO-LGBM model was 0.18, which was very high. Optimization improved the hybrid model’s performance compared to the default LGBM model.

As briefly discussed in Section 3.7, the a20-index is gaining widespread attention from researchers for demonstrating the accuracy of ML models. The a20-index indicates the percentage of model predictions that fall within a ±20% error margin. XGB and ACO-XGB achieved the highest a20-index values of 97.07% and 98.50%, respectively, signaling strong generalization and robustness in handling complex data relationships [18]. Furthermore, the a20-index for CB (97.01%) and ACO-CB (97.93%) showed that ACO achieved a very slight improvement in the predictive consistency of the CBR model. Also, GB (96.64%) and ACO-GB (98.01%) performed reasonably well. This indicates that ACO can minimize errors with fine-tuning. RF and ACO-RF demonstrated slightly lower accuracy, having a20 values of 95.89% and 96.27%, respectively. This may be due to their tendency to overfit during training. Furthermore, LGBM demonstrated the lowest a20-score (93.28%) due to greater fluctuations in its predictions. However, ACO-LGBM (95.14%) showed significant improvement in comparison to its non-optimized counterpart, highlighting the effectiveness of hyperparameter optimization by ACO.

#### Cross-Validation Analysis

To rigorously evaluate the generalization capability of the developed models, a 10-fold cross-validation (CV) framework was implemented. Unlike a single train–test split, CV systematically varies the training and validation subsets, thereby providing a more reliable estimate of model performance across different data distributions [84,85,86]. This approach mitigates the dependency on a specific data partition and reduces the risk of overfitting associated with a single split.

A 10-fold CV scheme was adopted as it provides a well-established balance between bias, variance, and computational efficiency. Using a smaller number of folds (e.g., 5-fold) can lead to higher bias due to reduced training data per fold, whereas a larger number of folds (e.g., leave-one-out cross-validation) may introduce higher variance and significantly increase computational cost. Therefore, the 10-fold strategy is particularly suitable for moderately sized datasets such as the present study (*n* = 1341).

The performance metrics were averaged across all folds, and the corresponding standard deviations were computed to quantify variability and model stability. The CV results are presented in Table 4. Among all models, the CatBoost (CB) model achieved the highest predictive accuracy, with an average R^2^ of 0.987 ± 0.007, and the lowest MAE and RMSE. The low standard deviation indicates strong consistency across folds, confirming the model’s robustness. Similarly, the ACO-GB (0.986 ± 0.009) and ACO-XGB (0.984 ± 0.010) models demonstrated competitive performance with low variability, indicating stable generalization behavior.

Although ACO-XGB exhibited superior performance in the train–test split (Section 4.2), the CV results reveal that CB and ACO-GB provided a more favorable balance between accuracy and stability. This highlights the importance of evaluating models beyond a single data split, as cross-validation captures variability induced by different training subsets.

In contrast, LGBM showed the weakest performance, with a significantly lower R^2^ (0.915 ± 0.050) and higher dispersion across folds, indicating poor generalization capability. The relatively higher variability observed in ACO-RF and ACO-LGBM also suggests sensitivity to data partitioning.

It is important to note that training-set performance metrics are not reported within the CV framework. In k-fold CV, each model is trained on different subsets of the data and evaluated on the remaining validation folds. Therefore, validation performance (mean ± standard deviation) provides an unbiased estimate of model generalization, whereas training metrics would not offer additional insight and may lead to overly optimistic interpretations.

The inclusion of standard deviation alongside mean performance metrics provides a quantitative measure of uncertainty, which is essential for assessing the reliability of machine learning models in engineering applications. These findings are further supported by the Monte Carlo simulation results (Section 4.5), in which CB and ACO-GB demonstrated more consistent performance with lower variability across varying input conditions. The observed overlap in performance ranges indicates that differences among top-performing models are not statistically distinct, but rather reflect variability arising from data partitioning and stochastic effects.

### 4.3. Feature Importance Analysis

**Figure 6:**
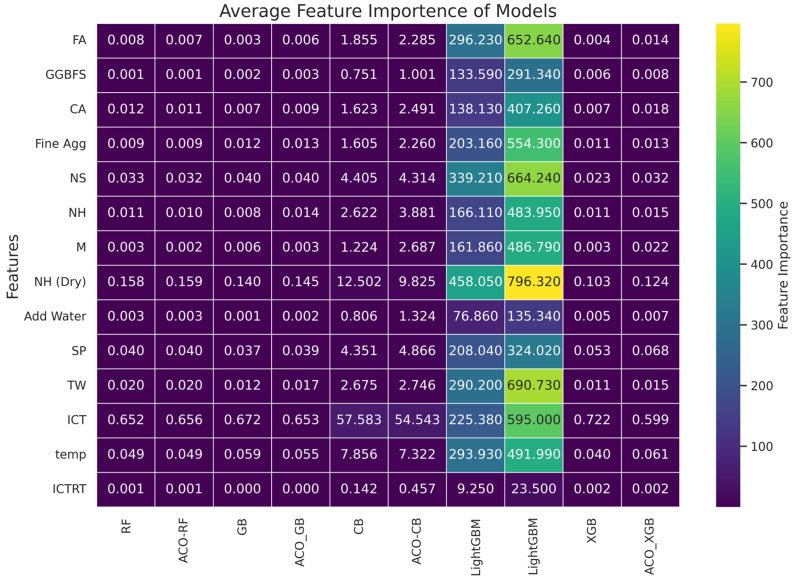
Distribution of feature importance across all the models.

To understand the decisions made by ML models, it is important to gain better insights into the input data and the models’ behavior. This need for transparency has created a new knowledge gap in interpreting and understanding data, ML models, and underlying patterns, as well as in formulating the estimation methodology. Similarly, this section aims to provide insights into the decision-making processes of the ML models used in this study to estimate carbon emissions from the production of FA-GGBFS-GPC, using feature importance analysis. This refers to assigning scores to input parameters used in developing prediction models. Figure 6 shows the feature importance for all ensemble ML models used in this study.

Across all ensemble ML models, ICT consistently emerged as the most influential feature. This highlights the critical role of ICT in predicting carbon emissions. Except for the LGBM model, ICT was the top contributor in carbon emissions in all models. This is because of the high energy demand for heat-producing appliances (e.g., oven). Similarly, NH (Dry) and temperature consistently ranked among the top contributors to carbon emissions in most ML models. Interestingly, the variation in the importance of various input features in different models, such as the lesser importance of ICT in LGBM and GGBFS in XGB, suggested that different models prioritize different aspects of data based on the intrinsic structure of algorithms.

Overall, ICT, NH (Dry), and temperature were significant predictors of carbon emissions across all models. However, the extent of their importance varied across the models, indicating the strengths and limitations of each algorithm. Since the adopted heuristic equation for CO_2_ emission estimation explicitly incorporates curing temperature, curing duration, and alkaline activator-related parameters, these variables inherently possess strong mathematical relationships with the target output. Consequently, the high predictive performance of the developed ML models should be interpreted with caution, as some of the models’ accuracy may stem from dependencies embedded in the emission calculation framework itself. Therefore, the developed models primarily reflect the capability of ensemble learning algorithms to reproduce complex nonlinear interactions within the adopted carbon emission estimation methodology rather than serving as fully independent lifecycle assessment tools. Future studies should utilize experimentally measured or comprehensive LCA-derived emission datasets to further evaluate the generalizability and independence of ML-based carbon emission prediction frameworks. A proper understanding of model-specific feature importances provides valuable feedback on the underlying estimation methodology. This will offer a more holistic view of the factors contributing to the carbon emissions of FA-GGBFS-GPC.

### 4.4. SHAP Explanation

To complement the feature importance analysis and obtain a more rigorous interpretation of model behavior, SHAP analysis was conducted on the ACO-GB model, given its demonstrated ability to balance stability and accuracy. SHAP is grounded in cooperative game theory and decomposes model predictions into additive contributions of individual input features, thereby enabling a consistent quantification of both the magnitude and direction of feature influence [87].

**Figure 7:**
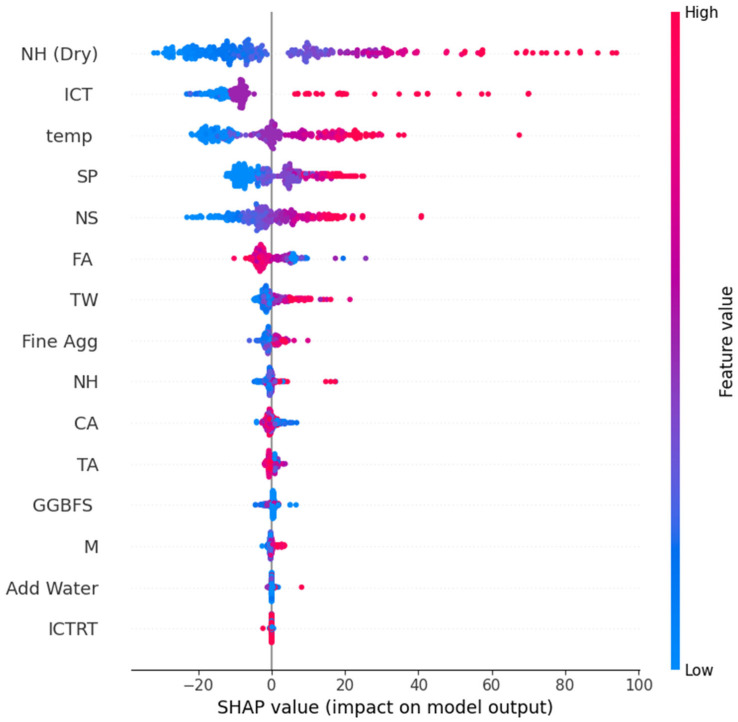
SHAP analysis for the ACO-GB model.

The SHAP summary plot (Figure 7) illustrates the distribution of feature contributions across the dataset. The results indicate that NH (Dry), ICT, and curing temperature have the greatest influence on predicted carbon emissions, consistent with the trends observed in the feature importance analysis. Specifically, higher values of these parameters are predominantly associated with positive SHAP values, indicating increased predicted emissions. This behavior is physically meaningful, as increased alkali activator content and extended thermal curing durations correspond to higher energy consumption and associated CO_2_ emissions during geopolymer production.

Furthermore, the dispersion of SHAP values for key features suggests nonlinear and interaction-driven effects in the dataset. For instance, the variation in SHAP values for ICT and NH (Dry) across different samples indicates that their impact on emissions is not uniform but depends on the combined influence of other process parameters. This observation highlights the ACO-GB model’s ability to capture complex, non-additive relationships inherent in FA-GGBFS-GPC systems.

The strong agreement between SHAP-derived feature contributions and the previously discussed feature importance rankings provides additional validation of the model’s internal consistency. At the same time, SHAP offers a more physically interpretable representation by explicitly linking variations in input parameters to changes in predicted emissions.

SHAP analysis was selectively applied to the best-performing model to avoid redundancy and maintain interpretational clarity, since models trained on the same dataset are expected to exhibit similar patterns of feature influence. Overall, the integration of SHAP-based interpretation enhances the transparency of the predictive framework and provides mechanistic insights into the key factors governing carbon emissions in geopolymer concrete.

While SHAP analysis provides insight into feature contributions and model behavior, it does not account for uncertainty in input parameters [88]. In practical applications, variability in material properties and curing conditions can influence prediction reliability. Therefore, Monte Carlo simulation and Gaussian white noise perturbation are performed in the subsequent section to evaluate model robustness under input uncertainty.

### 4.5. Model Stability Analysis Using Monte Carlo Simulation

Monte Carlo simulation (MCS) is a powerful approach to assessing the reliability and robustness of predictive models by running multiple repetitions across different scenarios [89,90]. In the present study, MCS with 100 independent runs was applied to evaluate the stability and accuracy of the developed ensemble learning models in varying conditions. Hyperparameter tuning, optimization techniques and random data sampling can bring fluctuations in model performance. MCS quantifies the consistency and model instability by performing multiple runs on the developed model.

The MCS analysis complements the cross-validation results by providing a stochastic assessment of model performance under input variability and uncertainty.

**Figure 8:**
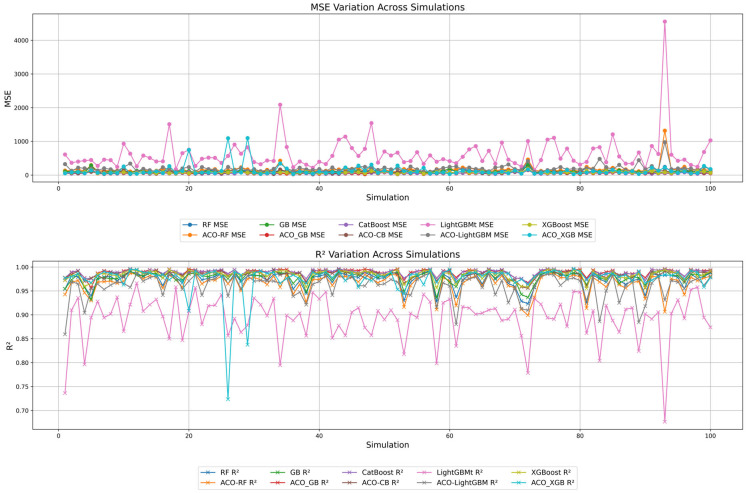
Variation in model performance in 100 runs of the Monte Carlo simulation.

To gauge model performance, two primary evaluation metrics are used: MSE and R^2^. MSE is a widely accepted statistical metric for measuring prediction errors, making it an effective indicator of model accuracy. In addition to MSE, R^2^ offers a clear representation of predictive power. These two metrics were chosen for their complementary perspectives. MSE indicates the magnitude of absolute error. However, R^2^ quantifies the explanatory strength of predictive models. Other statistical metrics were not chosen for this study because they do not add significant value beyond what MSE and R^2^ provide. Additionally, to further evaluate model stability in terms of accuracy and reliability, the standard deviations of MSE and R^2^ were calculated across 100 MCS runs. Figure 8 represents the variation in MSE and R^2^.

The results of MCS revealed that MSE was lower in the ACO-GB and CB models, at 52.42 and 54.85, respectively. In contrast, LGBM showed the highest MSE of 616.91, signaling substantial errors and a lack of model generalization. Ensemble models, without hyperparameter optimization, also showed satisfactory performance, but their predictive performance was not as good as the ACO-GB and CB models. Interestingly, the impact of hyperparameter optimization using ACO was dependent on the type of ML algorithm. ACO improved GB’s performance by reducing its MSE from 102.16 to 52.42. Conversely, it degraded the performance of the RF and XGB models, increasing their MSEs from 120.36 to 168.90 for RF and from 86.93 to 128.61 for XGB. These results indicate that ACO cannot improve the performance of all models and can cause model instability in some cases.

The R^2^ analysis for all models further supports these observations. CB and ACO-GB demonstrated reliability by achieving high R^2^ values, explaining almost 98% of the variance in the data. Despite this, LGBM showed weak predictive power, with the lowest R^2^ of 0.89. It was observed that ACO improved LGBM’s performance, increasing its R^2^ from 0.89 to 0.95, but it harmed RF and XGB. The R^2^ of RF decreased from 0.97 to 0.96, and similarly, XGB’s R^2^ decreased from 0.98 to 0.97. These observations indicate that while ACO can effectively optimize the hyperparameters of ML models, it may also impose a risk of instability in models by increasing their sensitivity to alterations in input data.

The stability of the developed ML models in the present study was also assessed using the standard deviations of MSE and R^2^. With the lowest MSE standard deviation, the CB model was the most stable. ACO-GB also demonstrated its robustness with a low MSE standard deviation of 20.36. In contrast, LGBM was highly unreliable, with the highest MSE standard deviation of 507.51, signaling extreme forecasting errors across different MCS runs. The same is observed in Figure 8, where the fluctuations are more pronounced for the LGBM model. Figure 8 shows similar variability in R^2^ trends. A minimal deviation in the standard deviation of R^2^ (0.006) was detected in the CB model [67]. Furthermore, the highest variation was observed in the ACO-XGB model, having an R^2^ standard deviation of 0.03. These observations indicate that ACO optimization is effective in improving accuracy. However, it can introduce uncertainty in the predictive performance of models, especially in the XGB model.

From the investigation of MCS results, a negative correlation between model accuracy and its stability was observed in some cases. In model performance assessment, XGB and ACO-XGB demonstrated high accuracy in predicting carbon emissions. However, these models showed substantial variation in performance across different MCS runs. This indicates that these models are highly sensitive and inconsistent, with variations in input data. This instability in XGB and ACO-XGB can be attributed to their boosting-based learning approach. Boosting models continuously correct the residuals of the previous model. This approach reduces bias but increases variance, making the model highly sensitive to small variations in the input dataset [72,73,74]. Moreover, the boosting approach is inherently sequential, which increases the risk of overfitting and reduces the model’s overall stability.

Interestingly, despite its moderate predictive accuracy, RF demonstrated greater stability than XGB and ACO-XGB. This can be understood through the model architecture. The aggregation approach of RF, which trains multiple trees on randomized data subsets, enhances stability by reducing the variance. This makes the performance RF model more consistent across MCS runs. Additionally, the averaging effect of RF regularizes the predictions naturally to avoid error propagation, thus reducing heavy deviations in predictions across different runs [91]. Moreover, RF has fewer hyperparameters than other ensemble models. This makes it less sensitive to hyperparameter changes, offering more robust performance. Altogether, the above-mentioned factors make RF a more stable model with moderate predictive performance in the present case.

LGBM demonstrated the least reliability due to poor accuracy and stability. It had the highest MSE across all MCS runs, with large fluctuations, signaling poor predictive performance. This can be explained by the aggressive pruning strategy adopted by LGBM. Although this strategy was developed to improve model efficiency, it can lead to overfitting and instability. Additionally, the LGBM model is more sensitive to outliers and to datasets with widely spaced data points, which may lead to inconsistent performance. Even after tuning hyperparameters with ACO, this model failed to demonstrate significant performance improvement. These observations reinforce the idea that hyperparameter optimization cannot compensate for the intrinsic flaws in model architecture.

From the above discussion, it can be concluded that CB and ACO-GB are the most suitable models for achieving high accuracy and stability, as they consistently yield minimal error and variability in predictions. ACO-XGB and XGB are highly accurate at predicting carbon emissions from FA-GGBFS-GPC [80], but they are less reliable in environments with expected data fluctuations. On the other hand, despite its moderate predictive performance, RF can be a feasible solution in cases where consistent, stable performance is required. LGBM is the least favorable model due to its poor stability and generalization.

Figure 8 represents the variation in R^2^ and MSE along 100 runs of MCS. It is evident from Figure 8 that the LGBM model exhibited the highest fluctuations in R^2^ and MSE. Further, despite high accuracy, ACO-XGB did not produce a stable model and exhibited slight variations. All other algorithms showed greater stability than ACO-XGB and LGBM, as discussed earlier in this section.

### 4.6. Gaussian White Noise

Gaussian white noise (GWN) plays a vital role in assessing the robustness and generalization ability of ML algorithms under real-world noisy data. GWN simulates real-world uncertainties in the dataset by introducing noise that a model may encounter during deployment. With GWN, we aim to explore models’ ability to maintain accuracy and stability in the presence of stochastic noise. This analysis will help identify model overfitting to specific data patterns and assess their generalization ability when handling noisy data. This study evaluates the impact of noise on model performance using four statistical performance metrics: MSE, RMSE, R^2^, and RSE [80,92]. These metrics are used to measure the model’s performance deviation in terms of accuracy and stability.

This analysis complements the cross-validation and Monte Carlo simulation results by providing an additional evaluation of model behavior under controlled noise perturbations.

**Figure 9:**
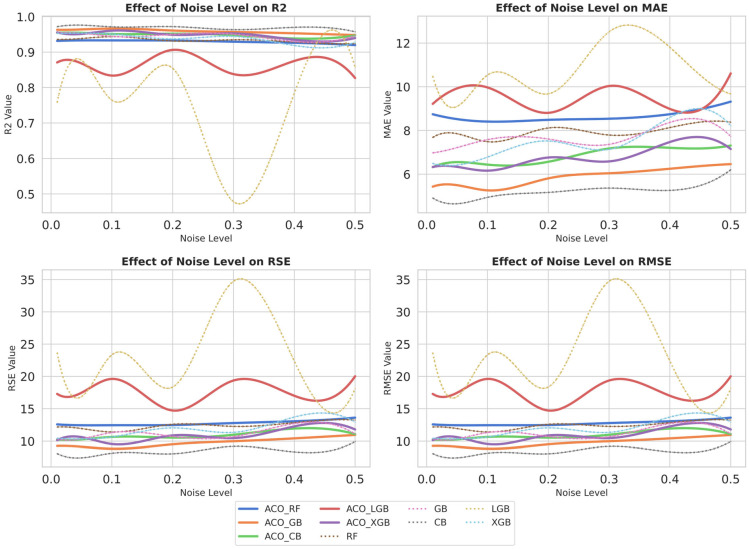
Effect of Gaussian white noise on various model parameters.

Figure 9 systematically presents the impact of GWN on the performance of ensemble and hybrid ensemble models, revealing significant variation. As the noise level increased, a general trend of performance deterioration was observed, with subsequent increases in MAE and RMSE and decreases in R^2^ values. Among all the models, the LGBM model demonstrated the highest sensitivity to noise, indicating the least robustness in handling noisy data. Furthermore, a comparative analysis of all models revealed that CB and ACO-GB demonstrated superior stability, consistently maintaining high R^2^ values and lower error metrics across all noise levels [93]. ACO-RF showed moderate resilience to noise, but performance slightly decreased as noise levels increased. ACO-XGB demonstrated the advantage of hyperparameter optimization and outperformed the default XGB, achieving higher R^2^ values while maintaining lower MAE and RMSE.

However, a noticeable increase in performance variability was observed in ACO-XGB at higher noise levels, indicating sensitivity to input perturbations.

Furthermore, patterns in Figure 9 indicate that hybrid models demonstrated more robust performance in the presence of noise by leveraging hyperparameter optimization. For instance, ACO-GB exhibited superior performance to the default GB model, underscoring the role of hyperparameter tuning in improving robustness to noise. These findings further highlight the role of nature-inspired optimization techniques, such as ACO, in effectively mitigating the adverse effects of noise, thereby enhancing the predictive stability of the models.

These results confirm the sensitivity of model performance to noise perturbations, with stability varying across model architectures.

## 5. Conclusions

This study developed and evaluated ensemble and hybrid-ensemble machine learning models to predict carbon emissions from fly ash- and GGBFS-based geopolymer concrete. A comprehensive dataset comprising 1341 data points was utilized to develop robust predictive relationships between mix design parameters, curing conditions, and the resulting carbon emissions of geopolymer concrete. Five ensemble learning algorithms, namely RF, GB, XGB, CB, and LGBM, along with their ACO-optimized counterparts, were assessed using multiple statistical indicators.

Among all developed models, ACO-XGB achieved the highest predictive accuracy, with a test R^2^ of 0.97 and low prediction errors, demonstrating its ability to effectively capture the nonlinear relationships governing carbon emissions. ACO-CB and ACO-GB also exhibited strong predictive performance and improved generalization compared with their standalone models. In contrast, LGBM showed the weakest performance despite improvement after hyperparameter optimization.

However, cross-validation and uncertainty analyses indicated that the performance differences among top models were relatively small, with CB and ACO-GB demonstrating more consistent and stable behavior under varying data and noise conditions.

Feature importance analysis revealed that initial curing time, dry sodium hydroxide content, and curing temperature were the dominant parameters influencing carbon emissions. Monte Carlo simulation and Gaussian white noise analyses further demonstrated that CB and ACO-GB exhibited greater robustness and stability under uncertain, noisy conditions, whereas XGB-based models, although highly accurate, were comparatively more sensitive to data fluctuations.

Taken together, the results demonstrate that model performance is governed by both predictive capability and sensitivity to data variability, emphasizing the need for balanced evaluation in engineering applications.

Overall, the findings confirm that hybrid ensemble learning integrated with nature-inspired optimization can provide reliable and efficient prediction of carbon emissions in geopolymer concrete. The proposed framework contributes to data-driven sustainable material design and may support the development of low-carbon construction materials. Future studies should investigate other optimization algorithms and deep learning approaches to further improve prediction reliability and model interpretability.

The developed predictive framework also demonstrates strong potential for practical implementation in sustainable construction and mix design optimization. By accurately estimating carbon emissions from various FA-GGBFS-GPC mixtures prior to production, the proposed ML models can assist engineers, researchers, and practitioners in selecting environmentally sustainable mix proportions that meet performance requirements. The feature importance analysis further provides practical guidance by identifying the most influential parameters affecting emissions, particularly initial curing time, curing temperature, and dry sodium hydroxide content. Such insights can support targeted optimization of curing regimes and activator dosages to reduce the environmental footprint of geopolymer concrete. In addition, the developed framework can be integrated into decision-support tools for sustainable material selection, enabling rapid evaluation of alternative mix designs without extensive experimental trials. Therefore, the proposed approach contributes not only to predictive modelling research but also to practical low-carbon construction strategies and sustainability-driven infrastructure development.

The reliability of the developed predictive framework was further validated through 10-fold cross-validation, which confirmed the consistency and generalization capability of the models across different data subsets. The low variability in performance metrics indicates that the models were not overfitted and can be effectively applied to unseen data. In addition, SHAP analysis provided a transparent interpretation of the model’s predictions by quantifying each input variable’s contribution. The results revealed that key parameters such as NH (dry), ICT, temp, SP and NS significantly influence the predicted output, thereby aligning with established physical and material behavior. This combination of robustness validation and interpretability strengthens the applicability of the proposed approach for practical engineering decision-making.

Future research should explore other optimization techniques, like particle swarm optimization, crow search optimization, genetic algorithms, etc., to further fine-tune the model performance. Furthermore, combining deep learning techniques such as convolutional neural networks (CNNs) and long short-term memory (LSTM) networks with ensemble models could improve model accuracy and adaptability. Furthermore, other uncertainty quantification approaches could facilitate the development of more interpretable models.

To support the mission of sustainable construction, future studies should delve deeper into multi-objective optimization approaches that minimize carbon emissions, improve mechanical properties, and ensure cost-effective, sustainable solutions. Finally, to bridge the gap between research and industrial applications, deploying ML models in real-time monitoring devices can be an effective solution. The findings in this study provide valuable insights into carbon emissions generated during the manufacturing of FA-GGBFS-GPC, thereby informing its contribution to global sustainability efforts and the production of environmentally friendly building materials.

## Figures and Tables

**Table 1:** Statistical description of the dataset.

Input Features	Mean	Std	Min	25%	50%	75%	Max
FA	326.44	143.32	0	263.5	381	416	640
GGBFS	85.83	145.95	0	0	0	144	560
CA	1121.91	173.50	525.40	1030.32	1167	1243	1591.34
Fine Agg	651.73	108.45	318.27	560.64	647.4	727	922.7
TA	1773.26	141.84	1207.80	1732.87	1823	1852.34	2112.19
NS	118.99	40.15	24.1	98.8	114.29	137.5	365
NH	61.53	25.88	9.64	45	56	70	232.18
M	11.48	3.24	2	8	12	14	23
NH (Dry)	27.48	15.28	2.97	18.86	23.29	30.06	130.02
Add Water	21.25	40.12	0	0	0	24.8	216
SP	5.47	7.19	0	0	4.3	7	48
TW	123.27	48.82	41.38	91.97	107.25	141.37	303.53
ICT	0.97	2.33	0	0	1	1	28
Temp	55.81	25.80	20	25	60	75	120
ICTRT	0.85	0.53	0	1	1	1	5
CO_2_ Emission	149.74	76.41	38.23	112.72	136.94	167.73	895.06

**Table 2:** List of optimized hyperparameters of all models.

Hyperparameters	RF	GBR	XGB	CBR	LGBM
*n*-estimators	203	1000	800	500	439
Learning rate	-	0.05	0.1	0.05	0.08
Maximum depth	9	4	6	10	7
Subsample	1	06	0.8	0.8	0.8
Minimum sample split	4	3	-	-	-
Minimum sample leaf	2	2	-	-	-
Minimum child weight	-	-	-	6	-
Minimum child samples	-	-	-	-	21
Alpha	-	0.8	-	-	0.04
Lambda	-	-	-	-	0.01

**Table 3:** Statistical metric performance of models.

Statistical Criteria		Models									
		RF	ACO-RF	GB	ACO-GB	XGB	ACO-XGB	CB	ACO-CB	LGBM	ACO-LGBM
R^2^	Train	0.96	0.97	0.97	0.99	0.98	0.99	0.97	0.99	0.97	0.98
	Test	0.89	0.93	0.95	0.96	0.96	0.97	0.94	0.97	0.73	0.81
MAE	Train	5.76	2.23	4.45	1.59	0.6	0.5	1.46	1.17	5.37	2.92
	Test	7.9	4.15	6.44	4.6	4.69	3.9	4.6	3.86	9.87	8.01
RSE	Train	0.008	0.003	0.006	0.001	0.001	0.001	0.001	0.001	0.023	0.003
	Test	0.045	0.038	0.040	0.031	0.023	0.023	0.043	0.038	0.263	0.182
RMSE	Train	7.39	4.88	6.51	2.58	1.68	1.63	2.46	1.77	13.03	5.15
	Test	11.23	10.68	9.71	8.46	7.64	6.17	7.81	6.45	24.69	20.55
a20	Test	95.89	96.27	96.64	98.01	97.07	98.5	97.01	97.93	93.28	95.14

**Table 4:** Model performance summary across 10-fold validation.

Statistical Criteria		Models									
		RF (Mean ± Std)	ACO-RF (Mean ± Std)	GB (Mean ± Std)	ACO-GB (Mean ± Std)	XGB (Mean ± Std)	ACO-XGB (Mean ± Std)	CB (Mean ± Std)	ACO-CB (Mean ± Std)	LGBM (Mean ± Std)	ACO-LGBM (Mean ± Std)
R^2^	Test	0.970 ± 0.018	0.958 ± 0.023	0.972 ± 0.016	0.987 ± 0.009	0.979 ± 0.012	0.984 ± 0.010	0.987 ± 0.007	0.980 ± 0.009	0.915 ± 0.050	0.958 ± 0.026
MAE	Test	5.17 ± 0.63	7.23 ± 1.04	5.88 ± 0.71	3.29 ± 0.50	4.04 ± 0.47	3.35 ± 0.45	3.21 ± 0.54	4.01 ± 1.12	8.26 ± 4.00	5.95 ± 01.96
RSE	Test	0.052 ± 0.006	0.041 ± 0.004	0.046 ± 0.007	0.036 ± 0.003	0.028 ± 0.008	0.032 ± 0.006	0.045 ± 0.003	0.043 ± 0.021	0.263 ± 0.163	0.186 ± 0.135
RMSE	Test	9.57 ± 1.68	11.60 ± 2.45	8.82 ± 1.42	6.38 ± 1.52	7.92 ± 1.17	6.84 ± 1.32	6.49 ± 1.90	8.93 ± 4.28	18.94 ± 14.60	12.00 ± 6.34
a20	Test	98.51 ± 1.27	97.73 ± 1.62	98.01 ± 0.62	98.03 ± 0.50	98.70 ± 0.60	98.2 ± 0.59	97.06 ± 0.40	97.78 ± 0.54	95.63 ± 1.64	96.5 ± 1.32

## Data Availability

The original contributions presented in the study are included in the article; further inquiries can be directed to the corresponding author.
